# Comprehensive genomic profiling for homologous recombination deficiency guides PARP inhibitor therapy recommendations in ovarian cancer

**DOI:** 10.3389/pore.2025.1612266

**Published:** 2025-11-13

**Authors:** Alp Inci, Isabell Witzel, Holger Moch, Sepehr Rahmani-Khajouei, Martin Zoche, Eleftherios Pierre Samartzis

**Affiliations:** 1 Department of Gynecology, University Hospital Zurich, University of Zurich, Zurich, Switzerland; 2 Department of Pathology and Molecular Pathology, University Hospital Zurich, University of Zurich, Zurich, Switzerland

**Keywords:** high-grade serous ovarian cancer, PARP inhibitors, homologous recombination deficiency, molecular profiling, precision oncology

## Abstract

**Objective:**

To evaluate the technical performance and clinical integration of FoundationOne®CDx (F1CDx) for high-grade serous ovarian cancer (HGSOC), focusing on its role in guiding PARP inhibitor (PARPi) therapy recommendations in a tertiary oncology center.

**Methods:**

We conducted a retrospective analysis of 178 F1CDx tests performed on 168 HGSOC patients with unknown BRCA mutation status between January 2019 and August 2024. Molecular findings, including *BRCA1/2* mutations, homologous recombination deficiency (HRD) status, loss of heterozygosity (LOH) scores, and HRR-related gene alterations, were correlated with tumor board recommendations and decisions for PARPi therapy. Laboratory turnaround time (TAT), assay performance, and integration into clinical workflows were assessed.

**Results:**

The F1CDx assay successfully generated comprehensive molecular profiles in 174 samples, with minimal limitations due to computational tumor content or inconclusive HRD readout. *BRCA1/2* mutations were detected in 13.1% of patients, and 39.5% of tumors were HRD-positive (LOH ≥16%). In the internal cohort, 81.8% of patients received PARPi therapy recommendations, all directly informed by F1CDx results. PARPi selection differed by HRD status, with niraparib favored in HR-proficient and olaparib in HRD-positive tumors. The mean laboratory TAT was 8.4 days (standard deviation ±3.8), with 92.2% of tests completed within 14 days. No additional profiling was required for PARPi therapy recommendation, and no incidental findings beyond the scope of HRD testing were detected.

**Conclusion:**

Molecular profiling with F1CDx proved to be a technically feasible, clinically impactful, and time-efficient assay, demonstrating its value in supporting molecular-guided PARPi therapy recommendations in the routine care of HGSOC patients.

## Introduction

High-grade serous ovarian cancer (HGSOC) is the most prevalent and deadliest subtype of epithelial ovarian cancer [1]. Despite advances in cytoreductive surgery, platinum-based chemotherapy, and maintenance strategies, the disease is associated with high recurrence rates and limited long-term survival [2, 3].

A key development in recent years has been the introduction of PARP inhibitors (PARPi) as maintenance therapy, particularly for patients with homologous recombination deficiency (HRD) [4–6]. HRD is defined by an impaired ability to repair DNA double-strand breaks through the homologous recombination repair (HRR) pathway. Approximately 50% of HGSOC cases harbor HRD [7], most commonly due to *BRCA1* or *BRCA2* mutations [8]. Additionally, alterations in other HRR-related genes – such as *RAD51C, RAD51D, PALB2,* and *CDK12 –* can also contribute to this phenotype, often referred to as “BRCAness” [9]. Altogether, mutations affecting the HRR pathway genes are reported in up to 30% of ovarian cancers [10]. As HRD is strongly associated with PARPi sensitivity, reliable detection of HRD has become a critical component of personalized treatment planning in HGSOC [11].

FoundationOne®CDx (F1CDx) by Foundation Medicine Inc. (FMI) is a prospectively validated, FDA-approved comprehensive genomic profiling (CGP) test that evaluates 324 cancer-related genes, including those implicated in HRD. The assay determines HRD status by detecting pathogenic *BRCA1/2* mutations and evaluating genomic loss of heterozygosity (LOH), using a clinically validated threshold of LOH ≥16% to define HRD [12] as evaluated in the ARIEL3 trial [6]. This copy number based algorithm can be used as a research use only HRDness biomarker [13]. While the analytical and clinical validity of F1CDx has been established in multiple tumor types, its impact in the specific context of HRD testing for PARPi therapy recommendations in HGSOC remains insufficiently characterized. Moreover, it is unclear whether the test consistently focuses on HRD-relevant alterations or occasionally detects variants of uncertain significance that could complicate clinical interpretation and decision-making regarding PARPi treatment decisions.

This study examines the implementation of F1CDx testing in routine clinical practice for patients with HGSOC at a tertiary academic center, with focus on whether the molecular results provided by the assay supported consistent and actionable PARPi therapy recommendations.

## Materials and methods

### Study design and patient population

This retrospective cohort study included all F1CDx (FMI, Cambridge, Massachusetts, USA) tests performed on patients with HGSOC at the University Hospital Zurich (Zurich, Switzerland) from 1 January 2019, to 31 August 2024. Eligible patients had a histopathologically confirmed diagnosis of high-grade serous carcinoma of the ovary, fallopian tube, or peritoneum (collectively termed ovarian cancer). All diagnoses were confirmed by board-certified pathologists. The censor date for clinical follow-up was 31 December 2024.

The study cohort was divided into two subgroups based on the origin of testing. Internal patients (39 patients, 40 samples) without known BRCA mutation status had F1CDx testing ordered by departments within the University Hospital Zurich. After excluding seven patients who did not provide informed consent for research participation, 32 patients (33 samples) were included for clinical evaluation. These patients received oncological care at our center and were discussed in two institutional tumor boards: the molecular tumor board and the gynecologic oncology tumor board. F1CDx test results were fully integrated into the decision-making process of both tumor boards and served as a basis for guiding PARPi treatment recommendations. External patients (136 patients, 145 samples) were referred from outside institutions solely for F1CDx testing. As these patients were managed externally and rarely discussed in our institutional tumor boards, the analysis in this group was limited to molecular findings reported by F1CDx, including *BRCA1/2* mutations, HRR-related gene alterations, LOH scores, and HRD status. Only internal patients were eligible for clinical follow-up and PARPi treatment evaluation.

In both subgroups, the total number of analyzed samples exceeded the number of individual patients because several patients underwent repeat F1CDx testing. In total, nine patients had more than one tumor sample analyzed. In these cases, a recurrent or metastatic lesion was tested in addition to the primary tumor to evaluate potential molecular evolution or to enable CGP when new tissue became available. One internal patient underwent duplicate testing on the same primary tumor block for technical confirmation of the HRD result.

Written informed consent was obtained from all participants for the use of their data. This study was performed in line with the principles of the Declaration of Helsinki and was approved by the Cantonal Ethics Committee of Zurich (BASEC reference number 2024-01536).

### Inclusion and exclusion criteria

The study population consisted of patients who had already met the predefined eligibility criteria: histopathologically confirmed HGSOC (at primary diagnosis or recurrence), unknown BRCA mutation status, availability of F1CDx results within defined study period, and written informed consent. No additional exclusion criteria were applied beyond the absence of consent.

In this context, “unknown BRCA mutation status” refers to the absence of a confirmed pathogenic *BRCA1/2* mutation within the F1CDx dataset. Internal patients may have undergone prior germline or limited somatic BRCA testing that yielded negative results; however, these results were not part of the present dataset, and F1CDx testing was performed to provide comprehensive HRD assessment, including LOH scoring.

### Sample processing and molecular profiling

Tissue specimens were collected through biopsy or surgical resection and preserved as formalin-fixed, paraffin-embedded (FFPE) blocks for molecular analysis. Internal samples followed institutional standards for tissue processing and storage. External samples were submitted via standard referral pathways. Genomic profiling was performed using the FDA-approved F1CDx assay, which evaluates a panel of 324 cancer-related genes. The assay detects single-nucleotide variants, small insertions and deletions, copy number variations, and structural variants. Furthermore, F1CDx assesses genomic signatures, including LOH, microsatellite instability, and tumor mutational burden. The assay uses hybrid capture technology combined with Illumina high-throughput sequencing by synthesis technology. Final reports – including LOH scores, HRD status, and HRR-related gene alterations – were reviewed and approved by board-certified molecular pathologists. FFPE processing was performed at the Molecular Tumor Profiling Center in Zurich, Switzerland, while bioinformatics analyses were conducted at FMI’s facility in Cambridge, Massachusetts, United States.

### Tumor content evaluation and loss of heterozygosity calculations

Board-certified pathologists at our institution estimated histologic tumor content (TC) prior to assay initiation. A minimum TC of 20% was required for F1CDx processing. In parallel, a computational TC (cTC) was calculated by FMI based on single nucleotide polymorphisms allele frequencies. The cTC provided by FMI influences the accuracy of genomic analyses, including copy number modeling and biomarker detection. Reliable LOH scoring and accurate copy number variations analysis require a cTC of at least 35%, as validated by FMI. When the cTC falls below this threshold, LOH scores are excluded from the final F1CDx report and labeled “cannot be determined”, with an explanatory note indicating insufficient cTC. Reasons for exclusion include low cTC, poor sample quality, noisy copy number data, or contamination. If re-evaluation results in a revised LOH score, the updated information is included in the final report and used in tumor board discussions. Genomic LOH was categorized as “high LOH” (≥16%), “low LOH” (<16%), or “LOH unknown” when calculation was not possible due to quality control issues.

### Data collection and variables evaluated

For internal patients, clinical data were collected on age, tumor stage, tumor location, type and extent of cytoreductive surgery, chemotherapy regimens, use of bevacizumab maintenance therapy, and PARPi therapy (agent, duration, reason for discontinuation). Molecular data extracted from F1CDx reports included *BRCA1/2* status, HRR gene alterations, LOH scores, HRD status, and cTC. For external patients, the same molecular variables were analyzed; however, clinical follow-up data were not available. Clinical information was extracted from the electronic medical management system KISIM (Cistec AG, Zurich, Switzerland) and anonymized prior to analysis. Efforts to address missing data included manual case-by-case review of electronic records and direct consultation with treating teams of internal patients.

### Statistical analysis

Statistical analyses were performed using IBM SPSS Statistics software (version 30.0, IBM Chicago, IL, United States). Descriptive statistics summarized patient and molecular characteristics. Continuous variables were reported as means with standard deviations (SD) and medians with interquartile ranges (IQR). Categorical variables were presented as frequencies and percentages. Group comparisons for categorical variables were assessed using Fisher’s Exact Test, including the association between the HRD status and the selected PARPi (niraparib or olaparib). Statistical significance was set at *p* < 0.05.

## Results

We analyzed 178 F1CDx tests performed on 168 patients with HGSOC between January 1, 2019, and August 31, 2024. This included 33 tests from 32 internal patients who received oncological care at our center and were discussed in multidisciplinary tumor boards, allowing for correlation of molecular findings with clinical decisions. The remaining 145 tests were conducted in 136 external patients, for whom only molecular data were available due to external management of care. We evaluated both the technical performance of F1CDx and its clinical relevance in therapy planning, with a particular focus on its ability to provide clinically interpretable results that inform PARPi therapy recommendations in a tertiary care setting.

### Clinical characteristics of the internal cohort

The demographic and clinicopathological characteristics of the 32 internal patients are presented in Table 1.

**TABLE 1:** Clinical characteristics and oncological treatment overview of internal patients.

Number of patients	32
Age (years) at diagnosis, median (range)	62 (37–84)
BMI (kg/m2) before surgery, mean (SD)	26 (4.6)
Stage at diagnosis, n (%)	
FIGO III	23 (71.9)
FIGO IV	9 (28.1)
Primary site, n (%)	
Ovary	21 (65.6)
Fallopian tube	8 (25.0)
Peritoneum	3 (9.4)
Debulking surgery at any time, n (%)	30 (93.8)
Of which PCS	18 (60.0)
Of which IDS	12 (40.0)
Total cycles of chemotherapy^a^, median (range)	6 (6–8)
Of which cycles of NACT, median (range)	3 (3–6)
Bevacizumab maintenance therapy, n (%)	20 (62.5)
Bulk of residual disease after surgery, n (%)	
No visible residual tumor/microscopical residual	20 (66.7)
Residual tumor ≤ 1 cm	6 (20)
Residual tumor > 1 cm	4 (13.3)

Data are shown as counts (percentage), mean (standard deviation) or median (range).

*BMI*: Body Mass Index. *FIGO:* International Federation of Gynecology and Obstetrics. *IDS:* interval debulking surgery. *NACT:* neoadjuvant chemotherapy. *PCS:* primary cytoreductive surgery. *SD:* standard deviation.

^a^ Only first-line chemotherapy with carboplatin and paclitaxel. All 32 patients received chemotherapy, of whom 12 received NACT.

### Molecular characteristics: homologous recombination deficiency and BRCA1/2 status

Among all 168 patients tested, 22 (13.1%) harbored pathogenic *BRCA1/2* mutations, including 16 *BRCA1-*mutated (9.5%) and 6 *BRCA2-*mutated (3.6%) cases. An additional 8 patients (4.8%) had mutations in non-BRCA HRR-related genes. HRD, defined as an LOH score ≥16%, was identified in 70 of 177 evaluable samples (39.5%), while the remaining 107 samples (60.5%) were HR proficient (HRP). One sample yielded inconclusive LOH results due to insufficient genomic signal. Among the *BRCA1/2-*mutated cases, 18 (82%) were HRD-positive (LOH ≥16%), and 4 (18%) were HRP (LOH <16%). All eight non-BRCA HRR-mutated tumors exhibited HRD-positive signatures. These findings confirm that most pathogenic HRR gene alterations co-occurred with high LOH scores, consistent with a functionally deficient HRR phenotype.

For internal patients with *BRCA1-*mutated tumors, germline testing was performed by the Institute of Medical Genetics, University of Zurich. One patient carried a confirmed germline *BRCA1* mutation (c.5266dup, p.Gln1756ProfsX74; LOH score: 29%), while the other had a somatic *BRCA1* mutation (c.1789G>T, pGlu597*; LOH score: 38%), detected only in the tumor tissue. Details of *BRCA1/2* mutations, other HRR gene alterations, and HRD status are summarized in Table 2.

**TABLE 2:** Overview of *BRCA1/2*, HRR gene mutations, and HRD status in HGSOC patients.

Number of patients *(n)*	Internal (32)	External (136)	Combined (168)
*BRCA 1/2* status			
*BRCA1* mut	2 (6.3)	14 (10.3)	16 (9.5)
*BRCA2* mut	0 (0.0)	6 (4.4)	6 (3.6)
*BRCA1/2* mut total^a^	2 (6.3)	20 (14.7)	22 (13.1)
*BRCA1/2* wt	30 (93.7)	116 (85.3)	146 (86.9)
Other HRR gene mutations			
*CDK12* mut	2 (6.3)	4 (2.9)	6 (3.6)
*RAD51C* mut	0 (0.0)	2 (1.5)	2 (1.2)
mut total	2 (6.3)	6 (4.4)	8 (4.8)

Number of samples *(n)*	Internal (33)	External (144)^b^	Combined (177)
HRD status			
HRP (LOH <16%)	22 (66.7)	85 (59.0)	107 (60.5)
HRD (LOH ≥16%)	11 (33.3)	59 (41.0)	70 (39.5)

Data shown for 177 tumor samples from 168 patients. HRD status was determined based on LOH score ≥16%. Data are shown as counts (percentage), unless stated otherwise. HRD status in BRCA1/2- and HRR-mutated cases is indicated in the text.

HGSOC: high-grade serous ovarian cancer. HRD: homologous recombination deficiency. HRP: homologous recombination proficiency. HRR: homologous recombination repair. LOH: loss of heterozygosity. mut: mutation. wt: wild-type.

^a^ Defined as both somatic and germline *BRCA1* or *BRCA2* mutations.

^b^ HRD status of one sample could not be determined.

### Impact of loss of heterozygosity and homologous recombination deficiency status on PARP inhibitor therapy planning

**FIGURE 1:**
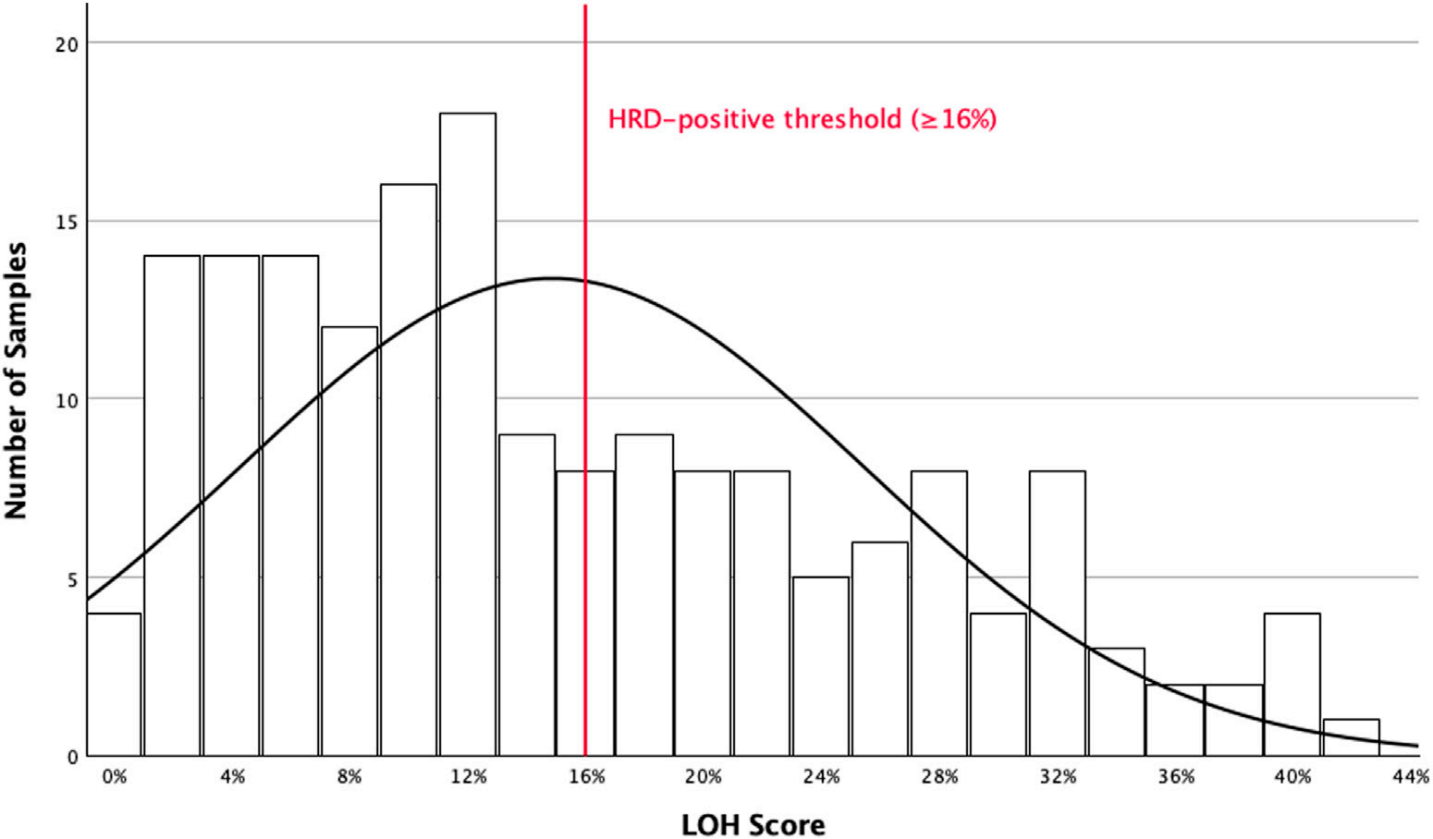
Distribution of LOH scores in HGSOC. Histogram showing the distribution of LOH scores in all HGSOC patients (*n* = 168) tested using the F1CDx assay. A total of 177 tumor samples were analyzed, of which 9 patients had two separate tumor samples tested. One sample was excluded due to inconclusive genomic results and classified as “LOH unknown.” F1CDx: FoundationOne®CDx. *HGSOC:* high-grade serous ovarian cancer. *HRD:* homologous recombination deficiency. *LOH:* loss of heterozygosity.

LOH scores were available for 177 of 178 tests. The mean LOH score was 14.9% (SD ±10.6), and the median was 12% (IQR: 6%–22%), with a range of 0%–42%. One sample was marked as “LOH unknown” due to inconclusive genomic readout. The overall distribution of LOH scores is shown in Figure 1.

Among internal patients, 27 of 33 tests (81.8%) resulted in a recommendation for PARPi therapy. In patients with LOH <16% (*n* = 22), 17 (77.3%) were recommended PARPi, while 5 (22.7%) were not. Among patients with LOH ≥16% (*n* = 11), 10 (90.9%) were recommended PARPi, and 1 (9.1%) was not. This latter case involved a patient tested twice from the same biopsy due to recurrence, where F1CDx was used to identify alternative actionable targets rather than to reassess HRD status. Excluding this case, all patients tested for HRD via F1CDx with an LOH ≥16% received a consistent recommendation for PARPi therapy.

### Concordance between tumor boards in PARP inhibitor therapy recommendations

All internal patients were discussed in both the molecular tumor board and gynecologic oncology tumor board. Concordant recommendations on PARPi therapy were observed in 29 of 33 cases (87.9%) regarding whether to recommend or withhold PARPi therapy. The molecular tumor board recommended PARPi in 23 cases (69.7%), while the gynecologic oncology tumor board did so in 27 cases (81.8%). Discrepancies (*n =* 4) (12%) occurred in two patients with HRD-negative tumors and intermediate LOH scores of 10% and 14%, and two patients with LOH scores of 11%, where interpretations differed regarding expected clinical benefit. In our cohort, F1CDx testing did not reveal any additional genetic alterations outside the intended HRR-related targets that were considered clinically relevant by the tumor boards. As a result, no further diagnostic steps – such as germline testing, imaging or invasive procedures – were initiated based on the testing results. Germline testing was conducted only in the two patients with *BRCA1-*mutated tumors, as part of standard care to determine mutation origin.

### PARP inhibitor selection: olaparib versus niraparib

Of the 27 patients for whom PARPi therapy was recommended, 24 had initiated treatment by the censor date. Olaparib and niraparib were prescribed in 12 patients each. Three patients had received a recommendation but had not yet started therapy at the time of censoring. PARPi selection was significantly associated with HRD status (*p* = 0.036): niraparib was more often chosen in HRP cases (10 of 14), whereas olaparib was preferred in HRD-positive patients (8 of 10). Both patients with *BRCA1* mutations received olaparib.

### Computational tumor content and loss of heterozygosity score reliability

The cTC values calculated by FMI for internal samples ranged from 9% to 94%, with a median of 60% (IQR: 44%–79%). Four cases had a cTC below 35%, the threshold required for reliable LOH scoring. In three of these samples (LOH scores: 0%, 2%, and 6%), LOH scores were excluded from the final F1CDx report due to low tumor purity and marked as “cannot be determined” with a note citing low cTC. In one patient, initially reported with a cTC of 20% and an LOH score of 19%, the LOH score was ultimately included in the final report after re-evaluation of the sequencing quality and genomic signal by FMI. This patient was later treated with niraparib. Details of patients with insufficient cTC for reliable LOH scoring and their associated PARPi decisions are shown in Table 3.

**TABLE 3:** Patients with cTC below the FMI validated threshold (<35%) for significant LOH scoring.

TC by FMI	LOH score	PARPi
9%^a^	0%	Not recommended
10%^a^	2%	Niraparib
20%	19%	Niraparib
21%^a^	6%	Olaparib

*FMI:* Foundation Medicine Inc. *LOH:* loss of heterozygosity. *PARPi:* PARP, inhibitor. *TC:* tumor content.

^a^ For three patients, the LOH scores were excluded from the final F1CDx report after re-evaluation due to low TC, and the clinicians were not informed of the LOH scores.

### Testing turnaround time

The testing turnaround time (TAT) was divided into five phases to illustrate each step in the testing process, as shown in Table 4. While the total TAT provides a comprehensive overview of the entire workflow, only the laboratory TAT – corresponding to the “Laboratory processing” phase – is under the direct control of the FMI laboratory conducting the genomic profiling. In our cohort, the laboratory TAT ranged from 0 to 20 days, with a mean of 8.4 days (SD ±3.8). A total of 92.2% of tests were completed within 14 days, meeting the laboratory’s guaranteed delivery timeline. The remaining phases are determined by external logistical and administrative factors beyond the laboratory’s control.

**TABLE 4:** F1CDx testing TAT across 177 tumor samples from 168 HGSOC patients.

Phase	Definition	Range (days)	Mean (days) (SD)	Distribution (samples, %)
Order processing	Time from order placement to sample receipt, including administrative processing and logistics	0–28	1.4 (4.0)	
Laboratory processing	Time from sample receipt to sequencing initiation, including sample preparation and quality control	0–20	8.4 (3.8)	≤7 days: 85 (47.8%) 8-10 days: 45 (25.3%) 11-14 days: 34 (19.1%) 15-21 days: 14 (7.9%) >21 days: 0 (0.0%)
Bioinformatics analysis	Time required for FMI’s bioinformatics pipeline to analyze sequencing data, including variant calling and report generation	0–17	2.6 (2.2)	
Report review and finalization	Time required for report generation, including review and approval by a pathologist	0–6	1.7 (1.4)	
Total turnaround time	Cumulative time from order placement to final report availability	5–39	14.1 (5.5)	

Percentages may not total 100 because of rounding.

*HGSOC:* high-grade serous ovarian cancer. *SD*: standard deviation. *TAT:* turnaround time.

## Discussion

This study provides a real-world evaluation of the technical performance, clinical applicability, and decision-making relevance of F1CDx for HRD testing in patients with HGSOC and unknown BRCA status. The assay consistently delivered interpretable molecular profiles, with only a small proportion of technically limited results. HRD status and LOH score could not be determined in one sample due to inconclusive genomic output, and in three patients, the cTC was below the FMI-validated threshold for reliable LOH scoring, limiting full HRD assessment. Despite these few limitations, the assay enabled timely and informed treatment planning in a multidisciplinary setting.

In the internal cohort, 81.8% of patients received a PARPi therapy recommendation, with treatment selection influenced by HRD status. HRD was detected in 39.5% of evaluable samples, and 13.1% of patients harbored pathogenic *BRCA1/2* mutations. The laboratory TAT was efficient, with a mean 8.4 ± 3.8 days and more than 90% of the results returned within the 14-day timeframe guaranteed by the laboratory. Importantly, no incidental findings unrelated to HRD testing led to additional diagnostic or follow-up procedures, supporting the assay’s focused clinical application in HRD-guided treatment planning.

The proportion of *BRCA1/2-*mutated cases in our cohort appears lower than that reported in larger population-based studies in HGSOC. This discrepancy likely reflects a preselection bias: at our center, F1CDx was frequently requested in patients who had previously tested negative for germline or somatic BRCA mutations. In contrast, patients with known BRCA mutations were commonly identified through earlier testing and thus less likely to undergo additional F1CDx testing, skewing the observed mutation prevalence. This context is essential for interpreting the *BRCA1/2* mutation rate in our study and does not indicate a limitation of the assay.

A further consideration in the interpretation of BRCA-related findings is the distinction between monoallelic and biallelic *BRCA1/2* inactivation. Only biallelic inactivation is reliably associated with HRD and PARPi sensitivity. The absence of a standardized method to differentiate mono-from biallelic loss in clinical reports complicates this assessment. In routine practice, clinicians may encounter patients with pathogenic BRCA mutations but negative HRD signatures, a discordance that can be explained by monoallelic alterations lacking functional HRD [13]. Our dataset did not allow for a definitive classification of allelic status, as this information is not included in the standard F1CDx report. Nonetheless, awareness of this distinction is critical for interpreting HRD testing results and counseling patients on PARPi efficacy. Moreover, BRCA homozygous deletions warrant particular attention. While infrequent, these events are highly predictive of sustained PARPi response, as tumors harboring homozygous BRCA deletions are less likely to develop reversion mutations – an established mechanism of acquired resistance. Recognizing such high-impact genomic events can inform long-term treatment strategies and highlights the added value of CGP.

Comparable studies evaluating other HRD testing platforms have reported similar HRD positivity rates and broader testing feasibility in clinical settings. However, longer median turnaround times and higher rates of tests failure due to insufficient tumor tissue have been observed [14]. While methodological differences and patient characteristics must be considered, our findings help contextualize the performance of F1CDx within the current landscape of HRD testing and underscore its suitability for integration into routine clinical use.

The F1CDx platform has been validated for CGP across multiple solid tumors [12]. Our study builds on this by offering real-world data on its specific utility in HGSOC, where HRD testing plays a central role in guiding maintenance therapy with PARPi. Unlike other HRD assays that focus solely on BRCA status or use single-method genomic scar signatures, F1CDx integrates *BRCA1/2* mutation status, non-BRCA HRR gene alterations, as well as LOH as a quantitative HRD indicator into a unified report. This integrated approach supports a more nuanced and clinically relevant assessment of HRD, aligning with previous studies that advocate for comprehensive HRD assessment in ovarian cancer [15, 16]. However, it should be acknowledged that in our study, the additional clinical benefit of CGP could not be conclusively demonstrated, as clinical outcomes such as progression-free or overall survival were not systematically assessed. Moreover, HRDsig – a copy number-based signature developed by FMI – was not included in our analysis, as it was not part of the clinical report during the study period and is currently designated as a research-use-only biomarker, not FDA-approved for clinical decision-making.

Our results also illustrate how F1CDx functioned as the primary genomic tool guiding PARPi therapy recommendations. Both tumor boards relied on the assay’s results to guide therapy recommendations, with high concordance (88%) in their decisions. In four discordant cases, the gynecologic oncology tumor board recommended niraparib despite HRD-negative or borderline LOH results. These recommendations highlight the application of the PRIMA trial [5], which demonstrated a clinically meaningful benefit of niraparib maintenance therapy irrespective of HRD status after response to platinum-based chemotherapy. At the time of these decisions, the PRIMA trial represented the current standard of care and was incorporated into international guidelines for PARPi inhibitor maintenance therapy. The patients concerned had borderline LOH scores – low but near-threshold values – platinum-sensitive disease, and no rapid progression, indicating a potentially intermediate HRD phenotype. The board therefore considered the presence of partial genomic instability, combined with favorable clinical conditions such as good performance status and preserved hematologic tolerance, justified PARPi use in analogy to PRIMA. The expectation of benefit in these borderline cases thus derived from both the clinical context and the evidence-based standard at that time, rather than from the HRD score alone. The data provided by F1CDx also directly informed PARPi selection, with niraparib more frequently recommended in HRP patients, and olaparib in HRD-positive and BRCA-mutated cases. These patterns reflect alignment with key clinical trials: niraparib selection was consistent with the PRIMA trial, which at that time supported its use regardless of HRD status, while olaparib use was guided by the PAOLA-1 [17] and SOLO-1 trials [18], which showed benefit in HRD-positive and BRCA-mutated populations.

This study has several limitations. Its retrospective design and relatively small size of the internal cohort limit the strength of correlations between clinical and molecular findings. Clinical data were available only for internal patients, while external cases were analyzed solely based on molecular data. F1CDx was implemented at our institution as the preferred HRD assay for HGSOC with unknown BRCA status in 2022, which contributed to the limited number of internal cases during the study period. Recent biomarkers such as HRDsig were not evaluated, as they were not in clinical use during the study period. As testing methodologies and treatment guidelines evolve, the relevance and applicability of our findings may be affected by future developments in HRD assessment and interpretation. Our conclusions reflect the testing and clinical practices in place between 2019 and 2024 and may differ from current or future standards.

In summary, we provide institutional-level insight into the feasibility and clinical relevance of CGP-based HRD testing for PARPi therapy planning in HGSOC. To our knowledge, this is among the first studies to assess how a commercially available, FDA-approved CGP assay can be successfully integrated into a multidisciplinary clinical workflow to support PARPi therapy decisions in HGSOC. Our study demonstrated that a single, validated broad-panel assay can reliably deliver integrated and clinically actionable HRD data within a timeframe compatible for tumor board discussions and therapeutic decision-making. Unlike testing approaches that provide fragmented information, F1CDx consolidates all relevant genomic inputs for HRD assessment – *BRCA1/2* status, HRR gene alterations, and LOH – into a single report, minimizing ambiguity and the need for further molecular workup. This efficiency is particularly valuable in high-volume oncology centers, where coordinated care and timely decision-making are critical. Future prospective studies are needed to assess the impact of F1CDx-guided HRD testing on clinical outcomes, long-term treatment benefit, and cost-effectiveness. Comparative studies between F1CDx and other HRD testing platforms would be especially useful for refining precision oncology strategies in ovarian cancer.

## Data Availability

The data analyzed in this study is subject to the following licenses/restrictions: The datasets analyzed in this study contain patient-level genomic and clinical information, which are considered sensitive personal health data. Due to patient privacy regulations, institutional policies, and restrictions from the Cantonal Ethics Committee of Zurich, these data cannot be made publicly available. Aggregated results are fully reported in the manuscript, and anonymized datasets may be made available from the corresponding author upon reasonable request, subject to ethical approval and institutional regulations. Requests to access these datasets should be directed to AI, alp.inci@usz.ch.
